# Association between delayed invasive blood pressure monitoring and all-cause mortality in intensive care unit patients with sepsis: a retrospective cohort study

**DOI:** 10.3389/fmed.2024.1446890

**Published:** 2024-11-06

**Authors:** Li Xiao, Pu Shen, Xue Han, Yi Yu

**Affiliations:** ^1^Department of Anesthesiology, The First Affiliated Hospital of Sun Yat-Sen University, Guangzhou, China; ^2^Department of Pain Medicine, The Second Affiliated Hospital of Guangzhou Medical University, Guangzhou Medical University, Guangzhou, China; ^3^Department of Critical Care Medicine, The Second Affiliated Hospital of Guangzhou University of Chinese Medicine, Guangzhou, Guangdong, China

**Keywords:** invasive blood pressure, intensive care unit, Sepsis, mortality, acute kidney injury

## Abstract

**Purpose:**

Haemodynamic management is essential in sepsis management. Invasive blood pressure (IBP) monitoring is the gold standard for blood pressure (BP) assessment. Here, we identified the most advantageous time frame for IBP monitoring to mitigate adverse outcomes in patients with sepsis.

**Methods:**

We included data on patients with sepsis from the Medical Information Mart for Intensive Care IV database. The primary endpoints comprised 28-and 90-day mortality rates, whereas secondary endpoints were acute kidney injury (AKI) rates and continuous renal replacement therapy (CRRT) requirement. To confirm our findings’ robustness, we performed multivariable Cox regression and logistic regression models, augmented by propensity score matching (PSM).

**Results:**

Of 18,326 patients hospitalised for sepsis, 9,056 (49.42%) and 9,270 (50.58%) were included in the early and delayed IBP-monitoring groups, respectively. Our multivariable Cox regression models revealed 20 and 21% significant increases in 28-and 90-day mortality in the delayed IBP monitoring group, respectively [hazard ratios (95% confidence intervals) = 1.20 (1.11–1.31) and 1.21 (1.12–1.31), respectively; both *p* < 0.001]. Moreover, significant increases were noted in AKI, CRRT and mechanical ventilation requirement risks in the delayed IBP monitoring group [odds ratios (95% confidence intervals) = 1.44 (1.34–1.56), 1.50 (1.26–1.78) and 1.79 (1.67–1.92), respectively; both *p* < 0.001]. PSM further confirmed the validity of our findings. Delayed IBP monitoring prolonged intensive care unit (ICU) stay without extending vasopressor use duration.

**Conclusion:**

Prolonged delay in IBP monitoring (≥3 h) may increase mortality risks in ICU patients with sepsis. Nevertheless, early IBP monitoring may reduce AKI, CRRT and mechanical ventilation requirement risks and shorten ICU stay. However, these results warrant further validation through randomised controlled trials.

## Introduction

Sepsis, characterised by dysregulated immune response following infection, remains a major public health challenge (1). Despite medical advancements in recent decades, sepsis mortality rates remain extremely high. The Surviving Sepsis Campaign (SSC) recommends blood pressure (BP) monitoring via arterial catheterisation for vasopressor-requiring patients as soon as feasible and when resources permit (2). However, Gershengorn et al. (3) reported that only 52% of vasopressor-receiving patients received arterial catheters during their stay in multiple intensive care units (ICUs) across the United States. In other words, approximately half of vasopressor-receiving patients may be managed through non-invasive BP (NIBP) monitoring rather than invasive BP (IBP) monitoring.

Peripheral arterial catheters, invasive haemodynamic monitoring devices used in critically ill patients, are regarded as the gold standard for BP monitoring. The placement of an arterial catheter enables safe, reliable and continuous measurement of arterial BP, facilitating real-time analysis of immediate, accurate BP information, based on which therapeutic decisions can be made (4). Despite the low risk of complications, arterial catheterisation is associated with some adverse effects, including ischemia, infection, thrombosis and increased healthcare costs due to frequent, often avoidable testing (5–7). Peripheral arterial catheters are essential for monitoring and assessing BP, arterial waveform beats in peripheral vascular resistance (8) and volume responsiveness (9)—all of which are evaluated in sepsis and septic shock cases. In the management of cardiovascular emergencies such as percutaneous coronary intervention and acute aortic dissection, accurate BP monitoring is essential for patient prognosis optimisation. IBP monitoring provides continuous, real-time BP data, aiding clinicians in making timely adjustments to treatment regimens and thus improving their patients’ clinical outcomes (10, 11).

According to the SSC guidelines, IBP monitoring should be established for patients requiring vasopressors as soon as possible; however, a specific timepoint is not recommended. However, direct evidence regarding the contributions of IBP monitoring to prognosis improvement or the optimal timing for IBP monitoring initiation in patients with sepsis remains scant. Moreover, no clinical study has indicated that delayed initiation of IBP monitoring increases mortality rates in patients with sepsis. Based on the currently available data, we hypothesised that delayed IBP monitoring increases sepsis-related mortality. Next, we conducted a retrospective study using 2008–2019 data from the Medical Information Mart for Intensive Care (MIMIC) IV database in order to investigate potential associations between delayed IBP monitoring and sepsis mortality.

## Methods

We included the data of patients with sepsis whose IBP measurements were available in the MIMIC-IV (version 2.2) database. This longitudinal, single-centre database includes data collected over 2008–2019 (12). One of the authors, YY, obtained permission to access the database (certificate ID: 6477678). This study adhered to the Guidelines for Strengthening the Reporting of Observational Studies in Epidemiology (13).

### Study population and data extraction

We only included patients aged ≥18 years and diagnosed as having sepsis (according to their discharge diagnoses). However, we excluded patients with repeated ICU admissions, aged <18 years, with missing IBP data, or with a < 24-h ICU stay. In cases of multiple ICU admissions, only the initial admission was considered. Demographic characteristics, vital signs, laboratory results, comorbidities, clinical severity scores, and other admission data were collected for each included patient. The diagnosis of sepsis was determined in accordance with the SSC guidelines (2).

### IBP

The patients who had IBP records during ICU stay were categorised as having the IBP monitoring time. We used a generalised additive model (GAM) to identify the nonlinear relationship. If a nonlinear correlation was observed, a two-piecewise linear regression model was conducted to calculate the threshold effect of the IBP monitoring time on mortality in terms of the smoothing plot.

### Covariates

The mortality risk factors associated with sepsis are documented elsewhere (14–16). The covariates comprised age, sex, body mass index (BMI), respiratory rate, temperature, oxygen saturation, white blood cell (WBC) count, haemoglobin level, haematocrit, platelet count, blood glucose level, office hours and weekdays. We also collected data regarding several health indicators, including sequential organ failure assessment (SOFA) score, as well as comorbidities, such as cardiovascular disease, kidney disease, liver disease, malignancy and neurological disease. We also extracted general information regarding ethnicity.

### Outcome

In this study, the primary outcome was sepsis-related mortality among patients with delayed IBP monitoring. Secondary outcomes included ICU stay duration, acute kidney injury (AKI) incidence, continuous renal replacement therapy (CRRT), mechanical ventilation (MV) requirement and vasopressor administration.

### Statistical analysis

Baseline characteristics of patients were distributed among different groups. Categorical data are presented as numbers (percentages), whereas continuous data are presented as means ± standard deviations or medians (interquartile ranges), as appropriate. Analysis of variance or rank sum testing was used to assess differences in continuous variables. Moreover, the chi-squared or Fisher exact test was employed to compare our study population’s characteristics across various outcome groups. We also applied consistent formatting and citation styles.

The missing data were replaced with the median because 5% of vital sign and laboratory parameter data were missing. A generalised additive model (GAM) was used to identify any nonlinear relationships (17). Survival outcomes were assessed through the construction of Kaplan–Meier survival curves and performance of log-rank analysis. We performed multivariate Cox regression analysis to evaluate specific correlations between delayed IBP and mortality, logistic regression analyses to assess the risk of AKI and CRRT and linear regression analyses to evaluate ICU-stay and vasoactive durations. To increase the rigour of our analysis, we performed propensity score matching (PSM) by using a 1:1 nearest neighbour matching algorithm with a calliper width of 0.1. The aforementioned variables were selected as covariates to generate propensity scores. The included covariates affected both the exposure factor and outcome variable. Moreover, we included variables occurring before the exposure factor and affecting the outcome variables significantly. To determine hazard ratios (HRs) for mortality with their 95% confidence intervals (CIs), we employed a multivariate Cox regression model with a robust variance estimator.

All statistical analyses were conducted using STATA (version 17.0), R packages (The R Foundation)^1^ and Free Statistics (version 1.8) (18). Multiple imputation was employed to account for missing values in Cox regression and model construction. Two-tailed *p* < 0.05 was considered to indicate statistical significance.

## Results

### Participants

**Figure 1:**
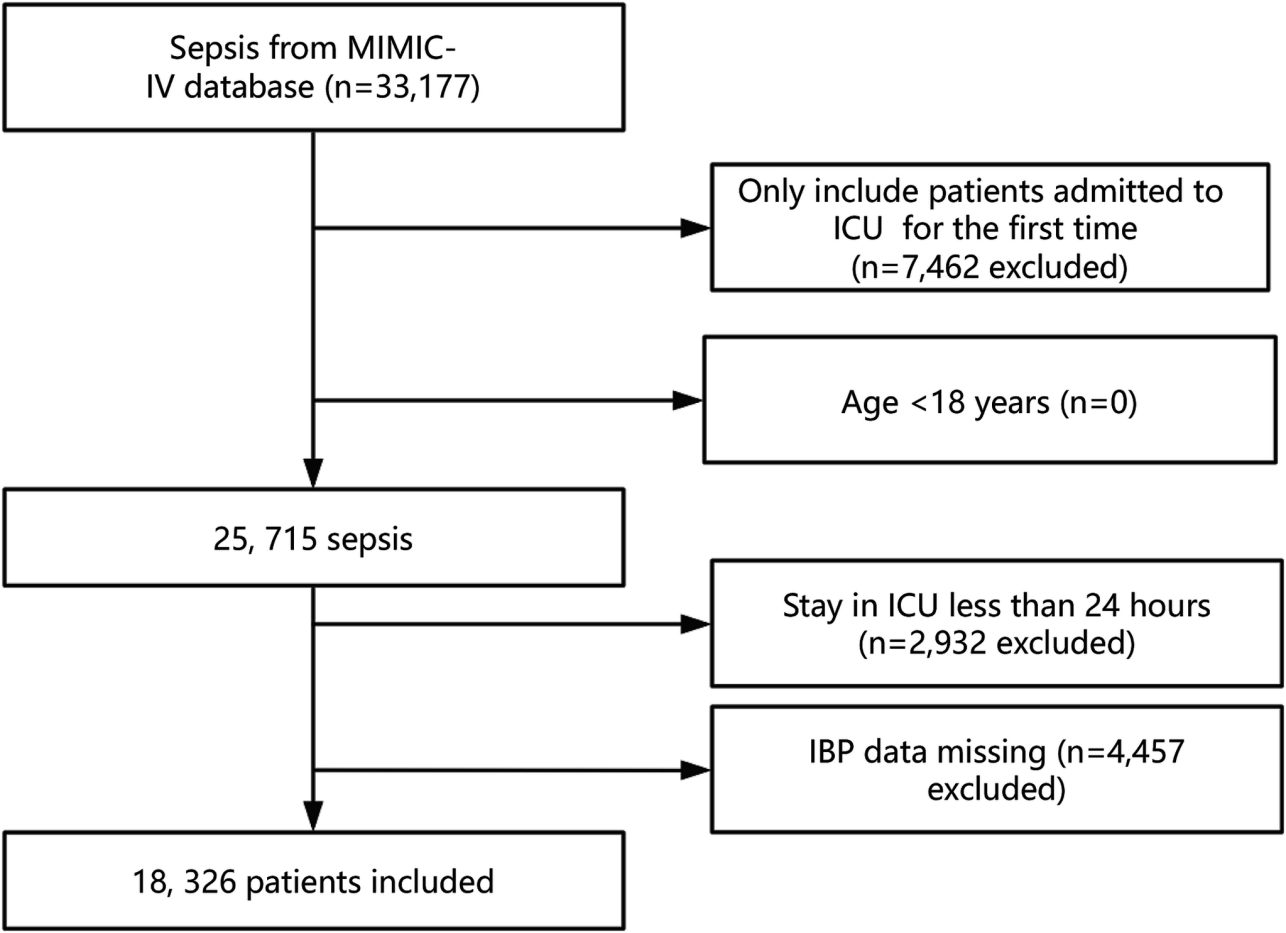
Participant selection process.

In total, 33,177 patients in the MIMIC-IV database fulfilled the criteria for sepsis. After consideration of our exclusion criteria (including removal of patients with repeated ICU admissions, aged <18 years, with missing IBP data, and with an ICU stay of <24 h), the final cohort comprised 18,326 patients with sepsis. Figure 1 presents our participant selection process.

### Nonlinear relationship of IBP monitoring with mortality

**Figure 2:**
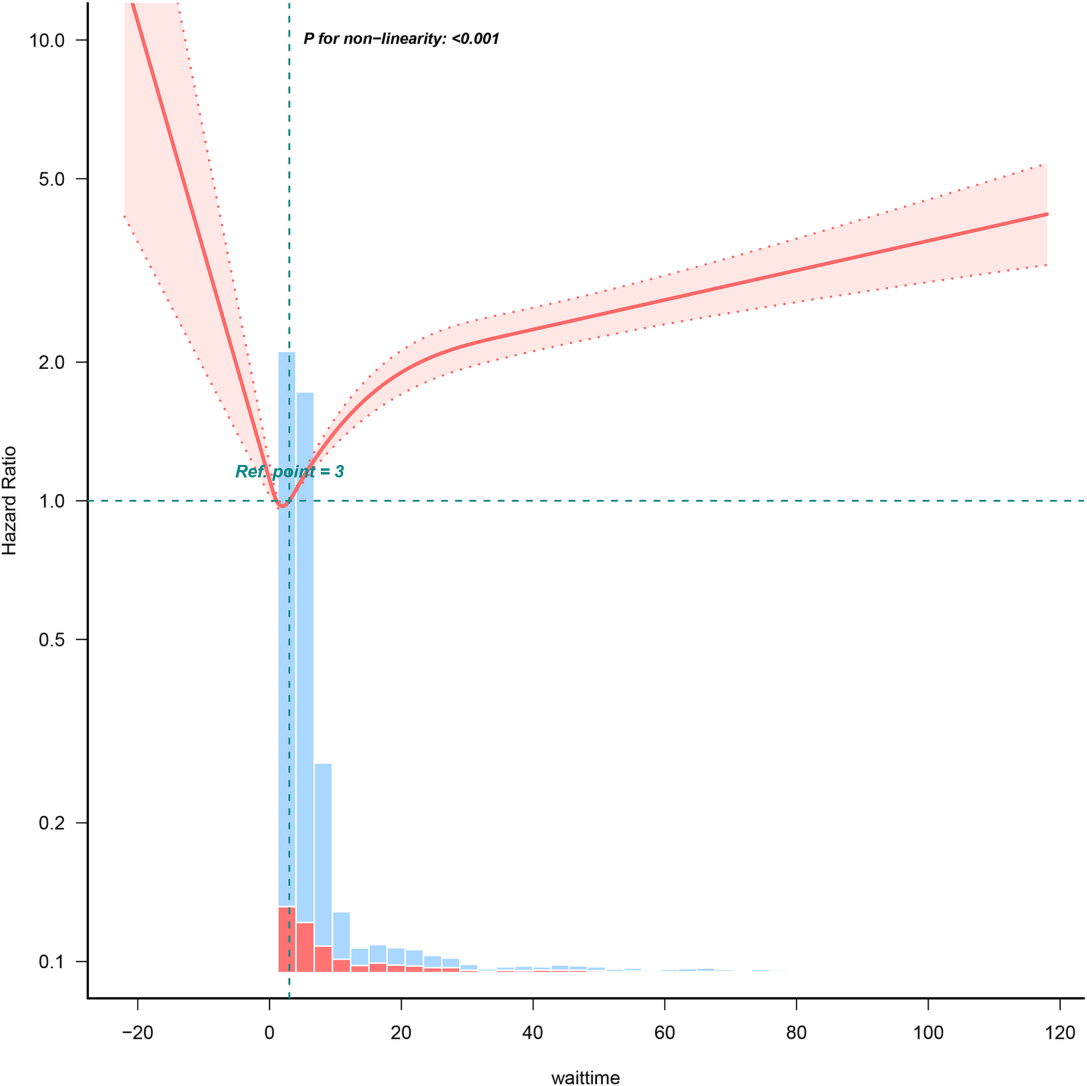
Relationship of delayed IBP monitoring and mortality. Data are adjusted for all covariates in Table 2.

A nonlinear association was noted between IBP monitoring delay and mortality. Next, by using a two-piecewise linear regression model, we identified that 3 h was the threshold for IBP monitoring delay. Prolonged delay in IBP monitoring (≥3 h) may increase mortality risks in ICU patients with sepsis (Figure 2).

### Baseline characteristics

Table 1 details our patients’ baseline characteristics. Of these patients (mean age = 64.1 ± 15.0 years; 59.8% male), 9,056 (49.42%) and 9,270 (50.58%) were included in the early and delayed IBP-monitoring groups, respectively. Compared with delayed IBP monitoring patients, those in the early IBP monitoring group tended to be younger, be female, have lower SOFA scores, demonstrate lower Charlson’s comorbidity index scores, have significantly lower 30-and 90-day mortality rates, and exhibit lower AKI and CRRT requirement risks.

**Table 1:** Baseline characteristics of our patients.

Variables	Total (*n* = 18,326)	Early IBP monitoring (<3 h; *n* = 9,056)	Delayed IBP monitoring (≥3 h; *n* = 9,270)	*p*
Age, years	64.1 ± 15.0	63.3 ± 15.3	64.9 ± 14.6	<0.001
Sex, male, n (%)	10,968 (59.8)	5,053 (55.8)	5,915 (63.8)	<0.001
BMI, kg/m^2^	28.9 ± 6.2	28.7 ± 6.0	29.1 ± 6.4	<0.001
Ethnicity, n (%)				0.95
White	13,465 (73.5)	6,652 (73.5)	6,813 (73.5)	
Others	4,861 (26.5)	2,404 (26.5)	2,457 (26.5)	
Insurance, n (%)				<0.001
Medicaid	1,082 (5.9)	562 (6.2)	520 (5.6)	
Medicare	7,681 (41.9)	3,610 (39.9)	4,071 (43.9)	
Others	9,563 (52.2)	4,884 (53.9)	4,679 (50.5)	
WBC, ×10^9^	13.1 ± 7.6	12.9 ± 6.1	13.3 ± 8.8	<0.001
Hb, g/L	10.8 ± 1.9	10.9 ± 1.8	10.7 ± 1.9	<0.001
HCT, %	32.4 ± 5.4	32.6 ± 5.3	32.1 ± 5.5	<0.001
PLT, ×10^9^	196.6 ± 95.0	202.5 ± 92.3	190.8 ± 97.2	<0.001
Respiration rate, bpm	18.6 ± 3.6	18.1 ± 3.4	19.0 ± 3.7	<0.001
Temperature, °C	36.8 ± 0.5	36.8 ± 0.5	36.8 ± 0.6	0.015
SpO2, %	97.2 ± 2.4	97.1 ± 2.6	97.3 ± 2.2	<0.001
Heart rate, bpm	83.9 ± 14.6	82.8 ± 14.3	85.0 ± 14.8	<0.001
MAP, mmHg	77.5 ± 9.7	78.6 ± 9.7	76.4 ± 9.6	<0.001
LAC, mmol/L	3.2 ± 2.6	3.2 ± 2.6	3.3 ± 2.6	0.001
BUN, median (IQR)	17.0 (12.5, 24.5)	16.0 (12.0, 22.5)	18.0 (13.0, 27.0)	<0.001
SCr, median (IQR)	0.9 (0.7, 1.2)	0.9 (0.7, 1.2)	0.9 (0.8, 1.3)	<0.001
Blood glucose, mmol/L	131 (118.5, 152.0)	131.3 (118.0, 154.0)	131 (119.0, 150.0)	0.163
Charlson’s comorbidity index	5.2 ± 2.7	5.0 ± 2.7	5.3 ± 2.6	<0.001
SOFA score	5.4 ± 3.6	4.7 ± 3.4	6.2 ± 3.6	<0.001
Myocardial infarct, n (%)	3,086 (16.8)	1,290 (14.2)	1,796 (19.4)	<0.001
Congestive heart failure, n (%)	4,024 (22.0)	1,666 (18.4)	2,358 (25.4)	<0.001
Cerebrovascular disease, n (%)	2,969 (16.2)	1,664 (18.4)	1,305 (14.1)	<0.001
Chronic pulmonary disease, n (%)	3,997 (21.8)	1,974 (21.8)	2,023 (21.8)	0.967
Diabetes, n (%)				<0.001
Complications	13,230 (72.2)	6,792 (75)	6,438 (69.4)	
Without complications	3,776 (20.6)	1,732 (19.1)	2,044 (22)	
With complications	1,320 (7.2)	532 (5.9)	788 (8.5)	
28-day mortality, n (%)	2,511 (13.7)	931 (10.3)	1,580 (17)	<0.001
90-day mortality, n (%)	2,741 (15.0)	1,001 (11.1)	1,740 (18.8)	<0.001
AKI in 7 days, n (%)	13,053 (71.2)	5,699 (62.9)	7,354 (79.3)	<0.001
CRRT, n (%)	1,053 (5.7)	335 (3.7)	718 (7.7)	<0.001

For each variable, values are reported as means ± standard deviations, medians (interquartile ranges), or numbers (percentages), as appropriate. IBP, invasive blood pressure; BMI, body mass index; WBC, white blood cells; Hb, haemoglobin; HCT, haematocrit; PLT, platelets; SpO2, pulse oxygen saturation; MAP, mean arterial pressure; LAC, lactate; BUN, blood urea nitrogen; SCr, serum creatinine; SOFA, sequential organ failure assessment score; AKI, acute kidney injury; CRRT, continuous renal replacement therapy; IQR, interquartile range.

### Relationship of IBP monitoring timing with mortality

**Figure 3:**
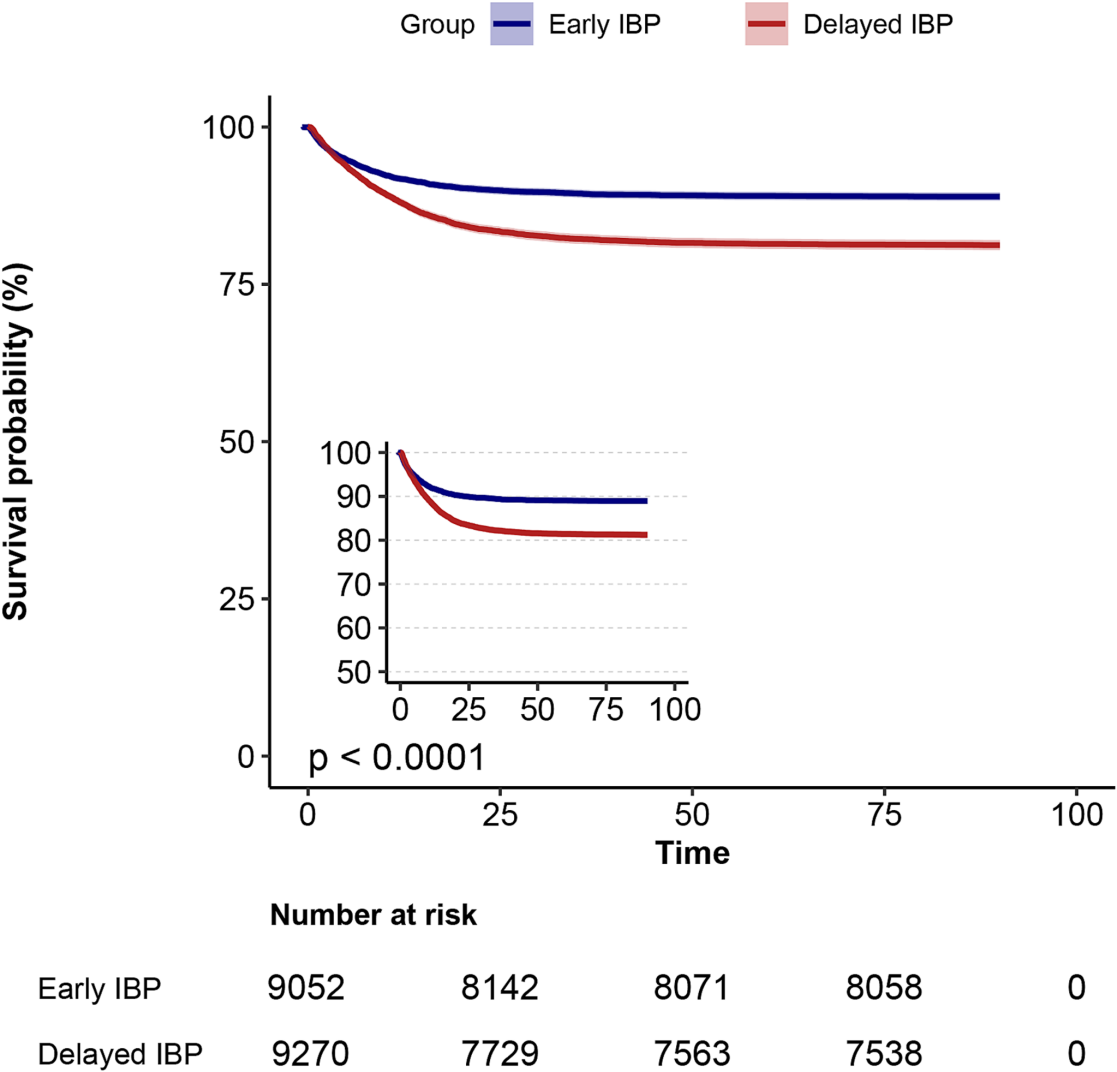
Kaplan–Meier survival curves of patients with sepsis at day 90, categorised based on IBP monitoring delay.

The results of our Kaplan–Meier curve analysis demonstrated a significant increase in 90-day mortality rates in the delayed IBP monitoring group than in the early IBP monitoring group (*p* < 0.0001, log-rank test; Figure 3).

In the univariate analysis of mortality risk, 28-and 90-day mortality rates were 70 and 75% higher in the delayed IBP monitoring group than in the early IBP monitoring group, respectively [HRs (95% CIs) = 1.7 (1.57–1.84) and 1.75 (1.62–1.89), respectively; both *p* < 0.001; Table 2]. In the extended multivariate Cox regression analysis (Table 2), we consistently observed significant HRs in the delayed IBP monitoring group across all models; the HRs ranged from 1.20 to 1.67 (both *p* < 0.001). After adjustments for all covariates outlined in Table 2, we observed 20 and 21% significant increases in 28-and 90-day mortality in the delayed IBP monitoring group, respectively [HRs (95% CIs) = 1.20 (1.11–1.31) and 1.21 (1.12–1.31), respectively; both *p* < 0.001, model 6]. These findings highlighted the robustness of our models.

**Table 2:** Effects of delayed IBP monitoring on mortality.

	28-day mortality			90-day mortality		
	HR for delayed IBP monitoring	95% CI	*p*	HR for delayed IBP monitoring	95% CI	*p*
Model 1	1.7	1.57–1.84	<0.001	1.75	1.62–1.89	<0.001
Model 2	1.67	1.54–1.82	<0.001	1.73	1.6–1.87	<0.001
Model 3	1.36	1.25–1.48	<0.001	1.39	1.29–1.51	<0.001
Model 4	1.35	1.24–1.47	<0.001	1.38	1.27–1.49	<0.001
Model 5	1.25	1.15–1.35	<0.001	1.27	1.17–1.37	<0.001
Model 6	1.20	1.11–1.31	<0.001	1.21	1.12–1.31	<0.001
PSM	1.21	1.10–1.33	<0.001	1.20	1.10–1.32	<0.001

Model 1: Not adjusted. Model 2: age, sex, ethnicity, and BMI. Model 3: Model 2 and heart rate, MAP, respiration rate, temperature, and SpO2. Model 4: Model 3 and lactate, glucose, WBC, HB, HCT, PLT, BUN, and SCr. Model 5: Model 4 and myocardial infarction, congestive heart failure, peripheral vascular disease, cerebrovascular disease, chronic pulmonary disease, diabetes, Charlson’s comorbidity index, and SOFA score. Model 6: Model 5 and hospital and ICU stay durations, mechanical ventilation, office hours, weekdays and CRRT requirement. PSM: Model 6. BMI, body mass index; WBC, white blood cells; Hb, haemoglobin; HCT, haematocrit; PLT, platelets; SpO2, pulse oxygen saturation; MAP, mean arterial pressure; LAC, lactate; BUN, blood urea nitrogen; SCr, serum creatinine; SOFA, sequential organ failure assessment score; AKI, acute kidney injury; CRRT, continuous renal replacement therapy; IQR, interquartile range; HR, hazard ratio; CI, confidence interval; PSM, propensity score matching.

### Relationship of delayed IBP monitoring with other outcomes

By incorporating all covariates listed in Table 3 in our models, the mean ICU stay was 13.11 h longer in the delayed IBP monitoring group than in the early IBP monitoring group [*β* (95% CI) = 13.11 (10.82–15.4)]. However, the mean duration of vasoactive drug use was only 1 h shorter in the delayed IBP monitoring group than in the early IBP monitoring group [β (95% CI) = −1.0 (−3.01 to 1.0); Table 4]. Moreover, the risks of AKI on day 7, CRRT and MV requirement were 44, 50 and 79% higher in the delayed IBP monitoring group than in the early IBP monitoring group, respectively [odds ratios (ORs; 95% CIs) = 1.44 (1.34–1.56), 1.50 (1.26–1.77) and 1.79 (1.67–1.92), respectively; Table 5].

**Table 3:** Effects of delayed IBP monitoring on ICU stay duration (in hours).

Variable	Model 1			Model 2		PSM		
	n. total	β (95% CI)	*p*	β (95% CI)	*p*	n. total	β (95% CI)	*p*
Early IBP monitoring	9,056	0 (Ref)		0(Ref)		6,888	0(Ref)	
Delayed IBP monitoring	9,270	41.37 (37.22–45.53)	<0.001	13.68 (11.07–16.29)	<0.001	6,888	4.2 (1.60–6.43)	0.001

**Table 4:** Effects of delayed IBP monitoring on vasoactive drug use duration (in hours).

Variable	Model 1			Model 2		PSM		
	n. total	β (95% CI)	*p*	β (95% CI)	*p*	n. total	β (95% CI)	*p*
Early IBP monitoring	9,056	0 (Ref)		0 (Ref)		6,888	0 (Ref)	
Delayed IBP monitoring	9,270	20.41 (17.62–23.21)	<0.001	−1.0 (−3.01 to 1)	0.117	6,888	−1.84 (−3.67 to 0.03)	0.051

Model 1: Not adjusted. Model 2: Age, sex, ethnicity, BMI, heart rate, MAP, respiration rate, temperature, SpO2, lactate, glucose, WBC, HB, HCT, PLT, BUN, SCr, myocardial infarction, congestive heart failure, peripheral vascular disease, cerebrovascular disease, chronic pulmonary disease, diabetes, Charlson’s comorbidity index, SOFA score, hospital and ICU stay durations, mechanical ventilation, office hours, weekdays and CRRT requirement. IBP, invasive blood pressure; BMI, body mass index; WBC, white blood cells; Hb, haemoglobin; HCT, haematocrit; PLT, platelets; SpO2, pulse oxygen saturation; MAP, mean arterial pressure; LAC, lactate; BUN, blood urea nitrogen; SCr, serum creatinine; SOFA, sequential organ failure assessment score; AKI, acute kidney injury; CRRT, continuous renal replacement therapy; IQR, interquartile range; CI, confidence interval; PSM, propensity score matching.

**Table 5:** Effects of delayed IBP monitoring on secondary outcomes.

	Model 1		Model 2	PSM	
	n. total	OR (95%CI)	OR (95%CI)	n. total	OR (95%CI)
AKI	9,056	2.26 (2.12–2.41)	1.44 (1.34–1.56)	6,888	1.31 (1.21–1.43)
CRRT	9,270	2.19 (1.91–2.5)	1.50 (1.26–1.78)	6,888	1.37 (1.14–1.65)
MV	10,888	2.48 (2.33–2.63)	1.79 (1.67–1.92)	8,491	1.58 (1.46–1.71)

AKI, acute kidney injury; CRRT, continuous renal replacement therapy; MV, mechanical ventilation.

### Subgroup and sensitivity analyses

Our findings exhibited robustness across all Cox regression models. After PSM, each group comprised 6,888 well-matched pairs, without significant between-group differences in the key indicators. Within the PSM cohort of 6,888 pairs, patients receiving early IBP monitoring demonstrated significantly lower 28-and 90-day mortality risks than those receiving delayed IBP monitoring [829 (12%) vs. 1,005 (14.6%) and 896 (13%) vs. 1,083 (15.7%), respectively; both *p* < 0.001]. Moreover, in our multivariate Cox regression analysis adjusted for all covariates, 28-and 90-day mortality rates were 21 and 20% higher in the delayed IBP monitoring group than in the early IBP monitoring group, respectively [HRs (95% CIs) = 1.21 (1.10–1.33) and 1.20 (1.10–1.32), respectively; both *p* < 0.001; Table 2]. This analysis also demonstrated that the risks of AKI on day 7, CRRT and MV requirement were 31, 37 and 58% higher in the delayed IBP monitoring group than in the early IBP monitoring group, respectively [odds ratios (ORs; 95% CIs) = 1.31 (1.21–1.43), 1.37 (1.14–1.65) and 1.58 (1.46–1.71), respectively; Table 5].

**Figure 4:**
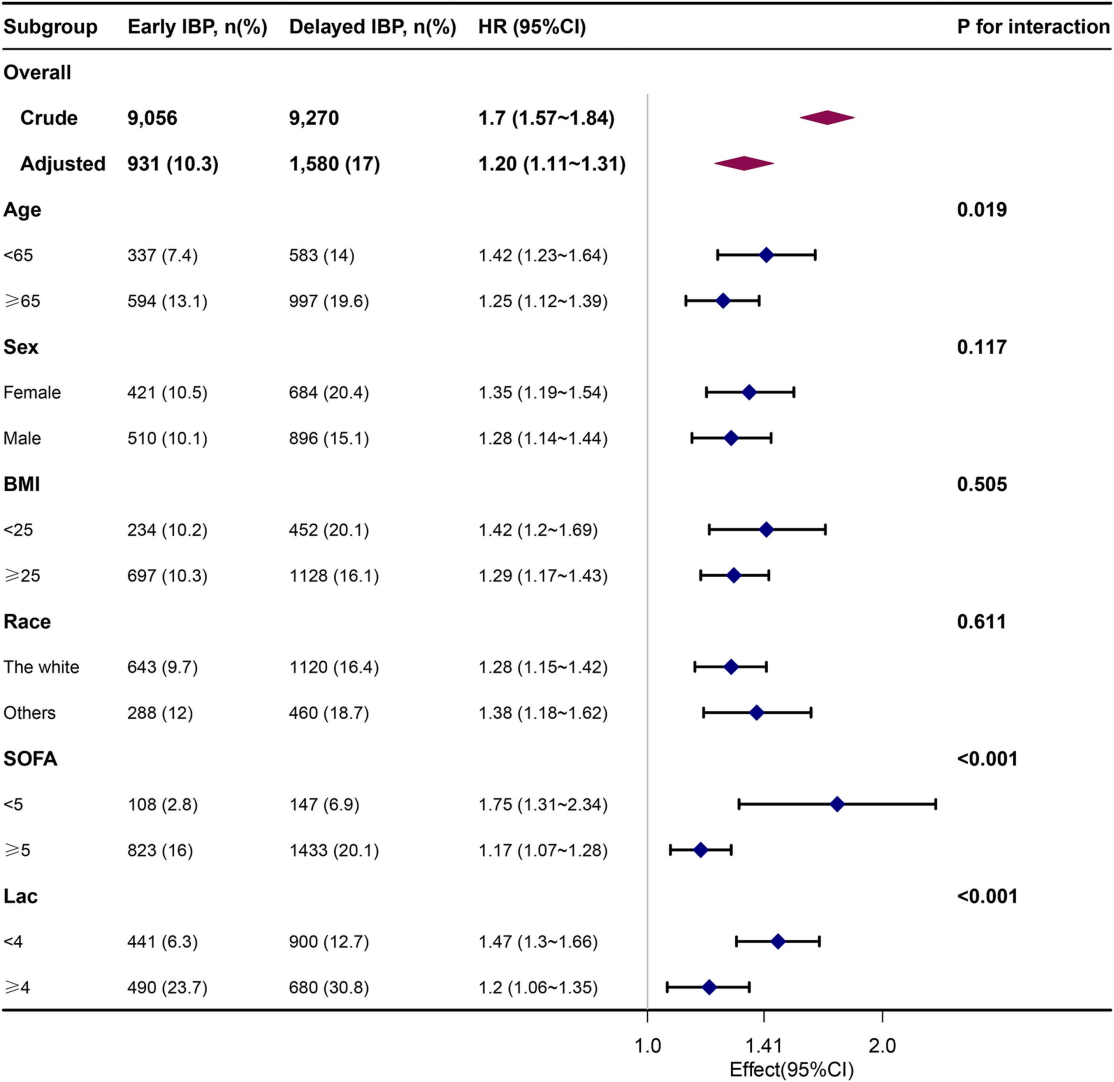
Association between delayed IBP monitoring and 28-day mortality according to baseline characteristics.

Our subgroup analyses indicated the continued robustness and reliability of the observed relationships. In particular, the adverse effect of delayed initiation of IBP was particularly notable in patients who were aged <65 years, had BMI ≥ 25 kg/m^2^, did not have kidney disease, SOFA scores <5, and lactate levels <4 mmol/L. No other significant interactions were observed within the subgroups (*P* for interaction >0.05; Figure 4). Similar trends were observed for 90-day mortality (Figure 4).

## Discussion

### Main result

In this study, we expanded on prior data regarding IBP monitoring use in patients with sepsis. By leveraging a comprehensive database, we obtained evidence confirming the beneficial effects of delayed IBP monitoring on mortality in patients with sepsis. Our outcomes indicated that delayed IBP monitoring is strongly correlated with a considerable increase in sepsis mortality. Moreover, through PSM, we increased the credibility of our conclusions, consistently highlighting the protective nature of early IBP in mitigating sepsis mortality risk.

### Effects of delayed IBP monitoring on mortality in patients with sepsis

IBP measurement is commonly used in the ICU because it enables precise beat-to-beat assessment of mean arterial pressure (MAP) (19). However, its correlation with mortality has been explored inadequately. IBP measurement also provides valuable insights into cardiac function, heart–lung interactions, the arterial system, and valvular diseases (8, 9, 20, 21). Numerous studies have highlighted the significance of IBP measurement in facilitating diagnosis and decision-making processes (22–24); however, the IBP measurement–mortality relationship in patients with sepsis remains poorly understood. In haemodynamically unstable critically ill patients, where critical decisions need to be made, IBP measurement is generally preferred over non-IBP methods (25). The benefits of arterial catheter use may outweigh the risks given the low complication rate and high BP measurement accuracy (26).

In the current study, rather than arbitrarily dividing patients into two groups, we categorised patients into early and delayed IBP monitoring groups to assess the time–dose–response relationship between delayed IBP monitoring and adjusted mortality. After adjustments for many confounding covariates, compared with patients who underwent delayed IBP monitoring (delayed IBP monitoring group), those who underwent IBP monitoring within 0–3 h (early IBP monitoring group) exhibited lower 28-and 90-day mortality rates in both the primary and PSM cohorts. By using precise time-to-IBP monitoring data, we empirically defined a threshold indicative of increased mortality risk. These findings highlighted the importance of early IBP measurement (≤3 h) in patients with sepsis. The current findings are consistent with those reported previously in this field. Compared with IBP monitoring, non-IBP monitoring is ineffective at guiding clinical care for paediatric critical illness management (27). In a clinical study on septic shock, noninvasive measurements of systolic and diastolic BP and MAP were significantly correlated with IBP measurements but not with non-IBP measurements (4). Nevertheless, some studies queried the necessity of IBP in sepsis or septic shock management. Studies have reported poor responsiveness of IBP measurements to clinical assessments, such as cardiac output and volume responsiveness prediction, particularly in patients with sepsis and septic shock; furthermore, IBP measurements do not appear to influence clinical decision-making strongly (28, 29).

### Relationship of delayed IBP monitoring with other outcomes

In ICU patients, AKI severity is strongly correlated with mortality, particularly in individuals with septic shock (30). The effects of BP on AKI incidence within this patient cohort remain under debate (31, 32). In a meta-analysis including five randomised controlled trials (RCTs) involving 1,485 patients, stringent BP management was linked to decreased postoperative AKI occurrence [OR (95% CI) = 0.73 (0.58–0.92); *p* = 0.007] (33). The aforementioned studies have indicated that hypotension is an independent risk factor for AKI. Early initiation of IBP monitoring can facilitate prompt alleviation of hypotension and maintenance of BP at an optimal level. The current findings corroborated these results: early IBP implementation reduces AKI and CRRT requirement risks.

The mechanisms underlying the association of early IBP monitoring with reduced mortality and AKI rates in patients with sepsis remain unclear. AKI has multiple aetiologies, such as prerenal factors. Prognostic enhancement in patients with AKI likely involves a multifaceted mechanism. Nevertheless, MAP is influenced by cardiac function, circulating blood volume and vascular tone; therefore, early MAP monitoring may facilitate prompt aetiological assessment and intervention.

Tissue perfusion pressure provides a visual representation of the desired BP level, enabling effective mortality prediction and ICU stay reduction (34). The difference in AKI incidence between patients undergoing non-IBP and IBP monitoring was nonsignificant (35). Additional studies assessing this aspect of the relationship of IBP monitoring with ICU stay length and vasoactive drug use duration and corroborating the current findings are warranted.

### Strengths and limitations

Our study has four primary strengths. First, we used a comprehensive, publicly available database, which ensured our data’s reliability and comprehensiveness. Second, investigation regarding the administration timepoint of IBP monitoring in patients with sepsis is limited; as such, relevant definitive conclusions remain unavailable. Here, we found that a significant delay in the initiation of IBP monitoring increases mortality risk substantially in patients with sepsis. IBP monitoring should ideally be initiated within 3 h of ICU admission. Our retrospective study compared mortality rates, ICU stays, and vasoactive medication use timings between the early and delayed IBP monitoring groups. The results provide a foundation for defining the timing of early and delayed IBP monitoring and the ideal sample size in subsequent RCTs. Third, we performed multiple sensitivity analyses to substantiate our results: (i) Cox regression analyses, adjusted using various models (to mitigate confounding effects), and (ii) PSM analysis; both methods yielded consistent results. This rigorous analytical approach supported the credibility and internal validity of our findings. Finally, considering the widespread use of IBP monitoring in critical care settings, our results have implications beyond the sepsis population.

This study also has a few limitations, which align with those typically encountered in observational research. First, retrospective studies are associated with inherent constraints. For instance, potential confounding variables may have remained unaccounted for in our study, whereas PSM aids in mitigating residual confounding, which tends to be a concern in observational studies. Moreover, our data lacked additional inflammation markers other than WBC count; thus, we could not eliminate the effects of selection and information biases completely. Second, our findings may not be generalisable because they were confined to a single country (the United States), a single database and a specific ICU setting. Nevertheless, our study included a substantially large, fairly representative sample. Subsequent multicentre prospective studies validating our results are warranted. Moreover, additional studies should investigate whether similar results occur in patients with other forms of critical illness. Third, many factors that influence sepsis-associated mortality risk—including educational attainment, smoking history, baseline cardiac function, hormone therapy, and history of medical conditions—are not available in the MIMIC-IV database. Fourth, Figure 2 illustrates that there was an increase in HR within the first three hours. The implementation of IBP monitoring within the initial three-hour period may be contingent upon a number of factors, including the transfer of patients from the operating theatre or other ICUs. This introduces a certain degree of uncertainty with regard to the outcome in this particular population. Given the multitude of potential confounding factors, it is not feasible to address these in a retrospective analysis. Accordingly, the conclusions of this article concentrate on investigating the impact of delayed IBP on mortality. As this finding requires further substantiation, we intend to investigate it in greater depth in a subsequent study. Finally, we could not elucidate the specific effects of IBP monitoring in individual patients because the MIMIC-IV database lacks data regarding IBP monitoring duration. Furthermore, IBP monitoring–related complications could not be analysed statistically because of missing data in the database. However, a systematic review reported that the overall pooled incidence of bloodstream infection is 0.96 per 1,000 catheter days (36).

## Conclusion

In ICU patients with sepsis, delayed IBP (≥3 h) monitoring initiation was associated with a greater risk of mortality rates. We recommend establishing IBP monitoring early (≤3 h) after ICU admission to improve patient outcomes, particularly in sepsis management. Additional RCTs substantiating the present results are warranted.

## Data Availability

The raw data supporting the conclusions of this article will be made available by the authors, without undue reservation.

## Footnotes

^1^http://www.R-project.org
